# Magnetic Resonance Spectroscopy of the Breast at 3T: Pre- and Post-Contrast Evaluation for Breast Lesion Characterization

**DOI:** 10.1100/2012/754380

**Published:** 2012-05-03

**Authors:** E. Kousi, I. Tsougos, K. Vasiou, K. Theodorou, A. Poultsidi, I. Fezoulidis, C. Kappas

**Affiliations:** ^1^Medical Physics Department, University of Thessaly, Biopolis, 41110 Larissa, Greece; ^2^Diagnostic Radiology Department, University of Thessaly, Biopolis, 41110 Larissa, Greece; ^3^Surgery Department, University of Thessaly, Biopolis, 41110 Larissa, Greece

## Abstract

*Purpose*. To determine whether in vivo proton magnetic resonance spectroscopy at 3T can provide accurate breast lesion characterization, and to determine the effect of gadolinium on the resonance of tCho. *Methods*. Twenty-four positive-mammogram patients were examined on a 3T MR scanner. 1H-MRS was performed before and after gadolinium administration. tCho peak was qualitatively evaluated before and after contrast injection. *Results*. Fourteen out of 27 lesions proved to be malignant after histopathological diagnosis. Using 1H-MRS, before contrast injection, 6/14 confirmed malignancies and 11/13 benign lesions were correctly classified; while, after contrast injection, 11/14 confirmed malignancies and 12/13 benign processes were correctly classified. Post gadolinium 1H-MRS proved useful in picking up tCho signal, improving the overall accuracy, sensitivity, and specificity by 35%, 83%, and 9%, respectively. *Conclusion*. 1H-MRS overall accuracy, sensitivity, and specificity in detecting breast lesion's malignancy were increased after gadolinium administration. It is prudent to perform 1H-MRS before contrast injection in large breast lesions to avoid choline underestimation. In cases of small or non-mass lesions, it is recommended to perform 1H-MRS after contrast injection for better voxel prescription to enable a reliable preoperative diagnosis.

## 1. Introduction

Conventional breast MRI has shown diagnostic sensitivities of 94–99% for screening breast lesions, whereas relatively low specificities have been reported, resulting in many unnecessary biopsies of benign lesions [1, 2]. Breast lesion characterization is based upon the combination of morphological features of the lesion and patterns of dynamic contrast enhancement of gadolinium-based contrast agent. However, as dynamic contrast-enhanced MR imaging has already gained acceptance as important breast imaging modality, it does not always lead to unambiguous diagnosis as their high sensitivity comes also with low specificity (53–80%) [3–7].

In addition to morphologic and kinetic analyses, molecular information has been expected to be useful for the diagnosis of breast disease. In vivo proton (1H) MR spectroscopy (MRS) of the breast, which provides molecular information obtained in a noninvasive manner, has shown that choline-containing compounds can be detected in most breast cancers [1, 2], whereas choline is generally not detectable in normal breast tissue.

The quality of 1H-MRS measurements-affecting Cho detectability depends on a variety of factors including MR sensitivity, spectral resolution, and voxel localization performance. Sensitivity increases linearly with voxel volume, increasing magnetic (B_0_) field strength, and the square root of the number of averages acquired in the MRS sequence. Spectral resolution is increased by higher B_0_ field strengths and by optimizing B_0_ shimming to improve MRI field homogeneity over a given region of interest (ROI) of a breast tumor.

Finally, effective placement of the voxel requires high-quality MR images to identify the breast lesion. The most straightforward use of the increased SNR in 3T MR imaging is for smaller pixel sizes (improved spatial resolution) and thinner slices or a combination of both. Thus, in breast spectroscopy, this gain in spatial resolution can be translated into a better adjustment of the size and position of the voxel on the lesion while minimizing adjacent fibrogranular or adipose tissue.

The aim of the most 1H-MRS studies reported to date has been to determine if the detectability of Cho resonance is indicative of malignancy. The working assumption for these studies is that higher tCho concentrations in malignancies make it easier to detect a Cho resonance. However, not all lesions are sufficiently large in size or may have non mass morphology limiting the diagnostic accuracy of the technique. Thus it is not always possible to identify the part of the lesion with the highest Cho concentration or reach acceptable levels of shimming due to inadequate voxel localization. Tozaki et al. mentioned that when 1H-MRS was applied to characterize mass lesions smaller than 15 mm, the diagnostic sensitivity in observing malignancy reached only 44%, whereas larger lesions increased diagnostic sensitivity to 82% [2].

To the direction of accurate voxel prescription, Lenkinski et al. mentioned that the majority of these in vivo 1H-MRS studies have been performed using single-voxel methods, where the lesion was first visualized on the postcontrast studies and the 1H-MRS voxel prescribed accordingly [8]. Those studies report that the presence of gadolinium-based contrast agents has a minor effect on the quality and peak intensities of MR spectra. Moreover, there are few in vivo studies determining the effects of gadolinium on the 1H-MRS on brain tumours that reported small changes (10–15%) in the Cho peak after contrast agent administration [9–14]. Similarly to those reports, Lenkinski et al. concluded that the negatively charged gadolinium chelates may lead to an underestimation of the levels of Cho present in breast lesions, since most studies use postcontrast 1H-MRS as it forms ion pairs with Cho, increasing the linewidth and dropping signal's height.

 Therefore, as 1H-MRS is continuously being incorporated to the clinical routine, it would be of interest to study the factors that influence the outcome of 1H-MRS and consequently propose an optimized 1H-MRS procedure for increased specificity in breast lesion characterization.

The present study focuses on the optimization of the 1H-MRS procedure during clinical practice in terms of choline detectability, pre- and postgadolinium injection.

## 2. Methods and Materials

### 2.1. Patients

 Twenty four patients (Mean ± S.D.;  53 ± 12  years) with suspicious mammograms were referred for MR evaluation. All patients were women, and none of them was a lactating mother. Lactating breasts are metabolically active, and there may be higher likelihood of them being positive for 1H-MRS investigations [15, 16]. All women underwent pre- and postcontrast 1H-MRS examination after given written informed consents. A total of 27 breast lesions were metabolically evaluated. Pathologic correlations were made for all patients.

### 2.2. MRI Acquisition

 All MRI and 1H-MRS scans were performed in the prone position for minimizing the effect of respiratory motion of the breasts, on a 3T MR scanner (GE, Healthcare, Signa HDx), using a dedicated phased array breast coil.

Conventional MRI protocol included axial T2-weighted Fast Spin Echo imaging sequence (T2-FSE, TR/TE = 3,600/100 msec, slice thickness = 4 mm, spacing = 0 mm), axial diffusion-weighted echo-planar imaging sequence (DW-EPI, TR/TE = 6,000/minimum msec, slice thickness = 4 mm, spacing = 0 mm), and axial short TI inversion recovery imaging sequence (STIR, TR/TE = 3,875/90 msec, slice thickness = 4 mm, spacing = 0 mm).

Dynamic contrast enhancement MRI sequence was performed using fat-suppressed three-dimensional T1-weigted vibrant dynamic images (flip angle = 10°, 1 mm^3^ isotropic voxel, one unenhanced and five contrast-enhanced acquisitions). Gadolinium was automatically injected over 10 seconds approximately.

### 2.3. 1H-MRS Protocol and Spectroscopic Data Analysis

 For 1H-MRS performance a single-voxel water and fat-suppressed point-resolved spectroscopy (PRESS) was acquired before and after contrast administration for evaluating the effect of neutral gadolinium chelates in MR spectra resonances.

The hypothesis was that the postcontrast localization of the voxel would yield a better consideration of the lesion morphology including as much of the lesion as possible while avoiding surrounding adipose tissue. Before 1H-MRS was performed, the channel contralateral to the lesion was automatically switched off. Automated parameter optimization consisted of frequency and receiver gain adjustment and gradient tuning. An automatic shimming adjustment was also performed to reach a full width at half maximum (FWHM) of the unsuppressed water peak lower than 25 Hz, as a quality parameter of the MR signal, 5 Hz lower than suggested by the manufacturer. For values greater than 25 Hz the automated shimming procedure was repeated. In the case of a value of FWHM greater than 25 Hz the voxel was re-adjusted to the region of interest. 1H-MRS sequence was acquired with the following technical parameters: TR/TE = 2,000/155 msec, number of excitations (NEX) = 56 for voxel size 3.375 cm^3^, and NEX = 32 for voxel size greater than 3.375 cm^3^. This relatively long TE was chosen to increase the visibility of tCho resonance because of the longer T2 of tCho (>350 msec) in comparison to that of lipids (~100 msec) [1]. Voxel size was chosen not less than 3.375 cm^3^ and not greater than 8 cm^3^, and it was carefully adjusted to the lesion. Moreover, a strong lipid and water spectral suppression was applied using a frequency-selective inversion pulse surrounded by a spoiler gradient pulse of opposite signs, which also incorporates a motion correction.

tCho resonance in breast spectra was qualitatively determined, and the criteria for determining the presence or absence of tCho were that a peak should be clearly identifiable at 3.2 ppm within the lesion.

## 3. Results

Table 1 summarizes the patient demographics, voxel size, and tCho presence or absence before and after contrast administration as well as histopathologic findings.

1H-MRS findings before contrast injection indicated that 6/14 confirmed malignancies and 11/13 benign lesions were correctly diagnosed (accuracy 62.9%, sensitivity 42.8%, and specificity 84.6%), whereas spectroscopy after contrast injection indicated 11/14 confirmed malignancies and 12/13 benign processes (accuracy 85.1%, sensitivity 78.5%, and specificity 92%). Accuracy, sensitivity, and specificity results are summarized at Table 2.

**Figure 1:**
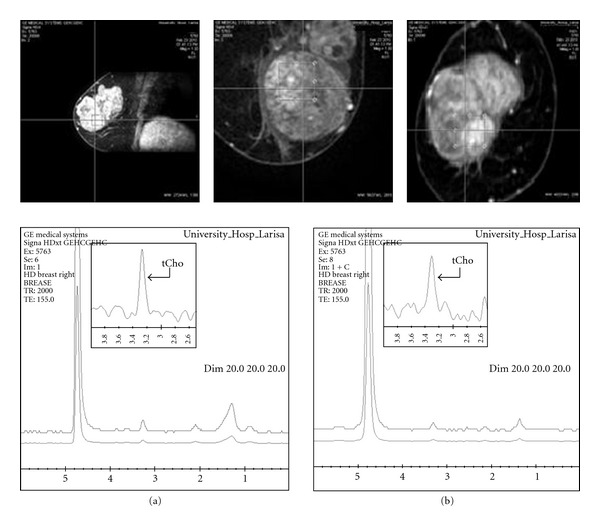
Breast MR spectra of a 60-year old patient, before (a) and 8 min after (b) contrast injection using a voxel of 14.7∗16.8∗15 cm^3^. The relative tCho peak intensity decreases, and line-width broadening in the postcontrast spectra is clearly visible.

**Figure 2:**
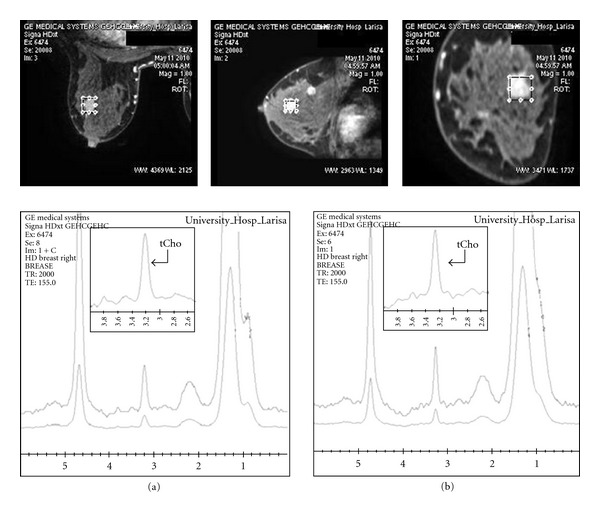
Breast MR spectra of a 49-year old patient, before (a) and 8 min after (b) contrast injection using a voxel of 20∗20∗20 cm^3^. The relative tCho peak intensity increase and line-width narrowing in the postcontrast spectra is clearly visible after better readjustment of the voxel on the lesion.

The effects of gadolinium chelates on tCho resonance after contrast administration are shown in Figures 1 and 2.  Figure 1 corresponds to an MRS evaluation in a sizeable malignant lesion while keeping the same size and position of the voxel before and after contrast injection. In this case, the administration of contrast caused mild-line broadening and decreased tCho peak's height without impeding malignancy assessment. This probably is a result of the direct contact of tCho with the contrast agent in order to form a complex, which leads to shortening of the relaxation times of the methyl protons of tCho through the electron-dipole interaction. 

On the contrary, when readjusted, the voxel in a smaller lesion, following the postcontrast image of the lesion, the line broadening, and the tCho peak's height, was further improved as suggestively is illustrated in Figure 2. Hence it is evident that, after contrast administration, a more adapted and accurate voxel localization upon the breast lesion derived better results. 

**Figure 3:**
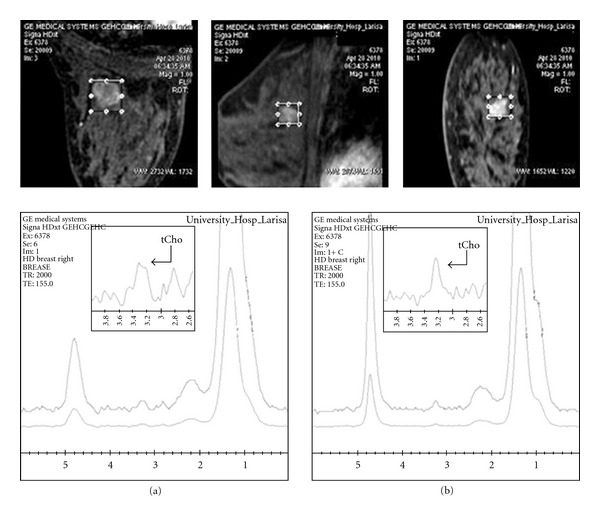
Breast MR spectra of a 35-year old patient with malignant breast lesion, before contrast injection (a) using a voxel of 15∗15∗15 cm^3^ and 8 min after (b) contrast injection using a voxel of 17∗16.3∗15 cm^3^ which is more adapted to the lesion's size. After contrast injection, tCho peak is clearly detectable.

Similarly, Figure 3 illustrates a case of a malignant breast lesion in which the selected voxel size of 15∗15∗15 cm^3^ before gadolinium injection did not adequately detect the tCho peak, and the lesion was misinterpreted as benign. However, after contrast administration, the lesion's delineation allowed a more accurate voxel prescription. The sensitivity of picking up adequate tCho signal from the lesion increased, as surrounded adipose tissue was avoided, and it is correctly diagnosed as malignant. This result was later histologically verified. 

**Figure 4:**
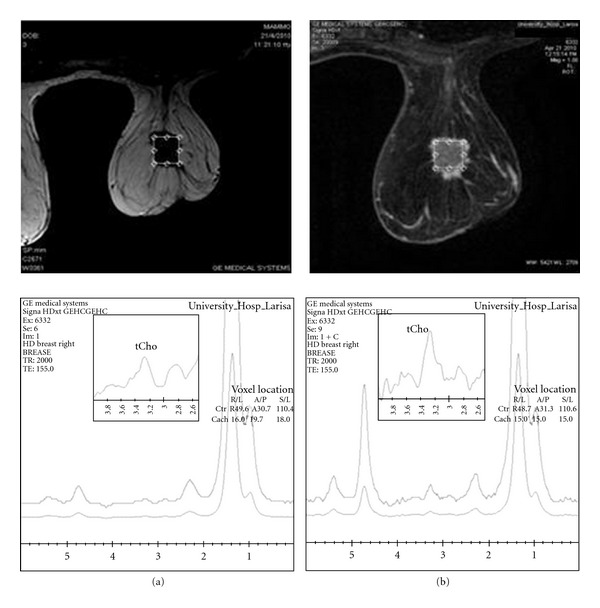
Breast MR spectra of a 63-years old patient with malignant breast lesion, before contrast injection (a) using a voxel of 18∗19.7∗18 cm^3^ and 8 min after (b) contrast injection using a voxel of 15∗15∗15 cm^3^ which is more adapted to the lesion's size. After contrast injection the overall spectral resolution is better as FWHM has reduced from 25 Hz to12 Hz.

 When adipose tissue that is not part of the pathologic process in breast cancer is included in the voxel for 1H-MRS, it reduces localized shimming, as its magnetic susceptibility differs from that of malignant tissues. In addition, the interaction between lipid signals and the pulsed gradients necessary for localization may produce lipid sideband artifacts, consequently causing spectral artifacts which may hinder interpretation of tCho signal [17]. As also shown in Figure 4, shimming significantly improved after contrast injection from 25 to 12 Hz. In that case, with the reduction of voxel size, the region of interest included greater amount of lesion than surrounding breast parenchyma; thus, field homogeneity allowed higher spectral resolution. 

The aforementioned results recommend that 1H-MRS accuracy, sensitivity, and specificity in detecting tCho resonance and hence lesion's malignancy are increased acquiring postcontrast spectra. The suggested reason for this improvement is the optimized lesion localization, resulting in better voxel positioning, exclusion of significant residual tissue signal, and thus increase of the signal-to-noise ratio of tCho signal and improvement of field homogeneity inside the region of interest. 

In order to characterize breast masses larger than 3.375 cm^3^ where a voxel of 1.5∗1.5∗1.5 cm^3^ or bigger can provide full lesion coverage, as that depicted in Figure 1, it is more prudent to perform 1H-MRS before contrast injection to allow the diagnostic resonance to be recorded, while maintaining the highest possible spectral resolution. 

However, when lesions are smaller or nonmass without clearly identifiable margins, it is recommended to perform 1H-MRS after contrast injection for better voxel prescription to enable an accurate and reliable preoperative diagnosis of such breast lesions (Figures 3 and 4).

## 4. Discussion 

As a biochemical measure of metabolism, 1H-MRS can detect cellular membrane turnover and proliferation by monitoring levels of a collection of chemicals with a choline base [18]. Elevated levels of the composite Cho signal (tCho) have been reported in many studies of excised human breast tumours, cultured human breast cancer cells, and animal models [16]. Fewer studies though have been devoted to the performance of in vivo 1H-MR spectroscopic detection of tCho in breast lesions [1–3, 10]. 

In this study, the effectiveness of clinical pre- and postcontrast 1H-MR spectroscopy in distinguishing between benign and malignant breast lesions at 3T was examined, and an optimized 1H-MRS procedure for increased accuracy, sensitivity, and specificity of detecting breast lesion malignancy was proposed. 

 Several parameters set during an automated 1H-MRS prescan procedure affect the effectiveness of the technique and typically include shimming, radiofrequency (RF) power calibrations, frequency adjustment, and voxel localization performance. 

Although all the prescan adjustments are important in the acquisition of high-quality in vivo MRS data, two parameters hold particular importance (shimming and voxel prescription), and thus it is crucial to spend the necessary time to achieve a well-shimmed region of interest (ROI), good water suppression, and thus high resolved spectra [19]. 

Variations in the main magnetic field (B_0_) that arise from extrinsic factors (notably susceptibility-induced field shifts) cause broadened and distorted peaks and must be minimized in order to acquire high-quality in vivo MRS data. Susceptibility-induced magnetic field distortions primarily arise from the different magnetic permeabilities. Consequently, when a subject is in a MR scanner, significant B_0_ inhomogeneities are generated that depend on the presence and distribution of different tissue types. These B_0_ inhomogeneities are often the dominant factor limiting successful MRS applications. 

The choice of voxel position and size is critical to achieve a good-quality diagnostic spectrum. Obviously it is important to locate the voxel in the appropriate area to detect the pathology under investigation. For example, within a breast lesion, there may be neoplastic, fibrogranular, or adipose tissue obscuring the choline resonance. 

Additionally, high-quality MR images are needed for accurate voxel prescription. In order to characterize a breast lesion, the voxel should be in the active tumour, but it can be difficult to distinguish these regions especially when the lesion is small or nonmass. As confirmed by this study, gadolinium has only a small effect on Cho peak (causing a small amount of line broadening), so postcontrast scans can be useful. As shown in Figures 2 and 3, after correction of voxel adjustment upon the breast lesion, the relative Cho peak intensity increased and linewidth became narrower in the postcontrast spectra. 

The fact that the linewidth of the residual Cho resonance in the presence of neutral gadolinium chelates increased (Figure 1) and the intensity of the tCho peak decreased, while maintaining voxel's size fixed, indicates that the area under the tCho curve remains unchangeable. As Lekinski et al. mentioned, these observations can only be explained by direct interactions of Cho with contrast agent rather than through any bulk susceptibility effects [8]. Whatever the explanation for the decrease in the levels of Cho that we observed, keeping the voxel fixed at pre- and postcontrast 1H-MRS, it seems that the gadolinium chelates may lead to a misinterpretation of the Cho present in human breast lesions. It is therefore advisable that if breast lesion is large enough to allow full coverage of a 3.375 cm^3^ voxel, then it is prudent to perform 1H-MRS before contrast injection, to avoid signal perturbation and choline underestimation. 

Our recommendation is also verified by the study of Baltzer et al. who reported that interaction of the contrast agent with Cho can cause false negative findings, especially in cases when signal-to-noise ratio of tCho is limited [20]. Therefore, they suggest that when Cho peak is clearly visible via precontrast 1H-MRS, it is recommended that it is more prudent to measure Cho SNR from precontrast spectra for avoiding signal misinterpretation and Cho concentration underestimation in cases of absolute quantification. 

On the other hand, during voxel prescription for 1H-MR spectroscopy, care should be taken to include as much of the lesion as possible while avoiding surrounding adipose tissue. In this study it was verified that before contrast administration, breast lesion margins are often not clearly identified especially in dense breast parenchyma. Therefore, the voxel size could be selected quite large or eccentric in respect to lesion size and location, respectively; hence Cho could become undetectable. To avoid false-negativity in the characterization of breast lesions, it is important to localize the voxel after contrast injection, for accurate voxel assessment. As illustrated in Figure 2, it is suggested that, after contrast administration, the more careful consideration of lesion morphology and enhancement kinetics allowed more accurate voxel localization upon the breast lesion. This derived less field inhomogeneity inside the selected voxel (Figure 4) resulting in high-quality spectra and thus higher overall accuracy, sensitivity, and sensitivity in detecting lesion's malignancy. Taking into account that the presence of gadolinium itself does not significantly affect the Cho signal, our findings are in agreement with studies supporting that the lesional size is one of the main issues which must be ensured in the performance of clinical in vivo breast 1H-MRS for picking up adequate amount of tCho signal [16, 17]. Thus, the likelihood to differentiate between malignant and benign breast lesions is increased using postcontrast 1H-MRS techniques. 

Furthermore, variations in the main magnetic field (B_0_) that arise from different magnetic permeability of fat and lesion tissue interface cause broadening and distortion of spectrum resonance characteristics and must be minimized to acquire high-quality in vivo MR spectra [17]. The aforementioned observations are also confirmed from the present study, with improved overall accuracy, sensitivity, and specificity of 1H-MRS technique in detecting lesion's malignancy after contrast agent administration as the region of interest was more accurately defined. Thus, it is suggested that, in order to exclude significant fatty tissue signal contribution in the assessed volume of interest, 1H-MRS should be performed after gadolinium injection. Exclusion of adipose tissue from the voxel lead is to correct tCho sampling of the lesion and derives better shimming results and better spectral resolution, as depicted in figures 3 and 4, respectively, in concordance with Yeh [21]. 

Considering that the 1H-MRS signal is an average of multiple signals acquired in possibly different breathing phases, without a frequency correction, both the peak position and height, as well as the peak SNR are infected. Therefore, any improvement in the signal-to-noise ratio that will effectively enhance the detection of tCho signal may further increase the specificity and sensitivity, improving the diagnostic performance of breast 1H-MRS [17, 21]. 

## 5. Conclusion

1H-MRS can be very useful in the detection and differentiation of malignant from nonmalignant breast lesions and can be implemented as an invaluable tool in the clinical setting. Furthermore our results recommend that 1H-MRS accuracy, sensitivity, and specificity in detecting tCho resonance and hence lesion's malignancy of breast lesions are increased when acquiring postcontrast spectra. The suggested reason for this improvement is the increase of the signal-to-noise ratio of tCho signal and the improvement of field homogeneity inside the region of interest, which is caused by the optimized voxel positioning on the lesion, resulting in the exclusion of significant residual tissue signal contribution in the assessed volume of interest. 

However, gadolinium-based contrast agents can have from minor to stronger effects on tCho detection in postcontrast 1H-MRS. It is therefore advisable that if breast lesion is large enough to allow accurate detection and full coverage of a >3.375 cm^3^ voxel (15 × 15 × 15 mm^3^), then it is prudent to perform 1H-MRS before contrast injection, to avoid signal perturbation and choline underestimation. On the other hand in cases of small or not well-oriented lesions, it is recommended to perform 1H-MRS after contrast injection for better voxel prescription to enable an accurate and reliable preoperative diagnosis. 

We strongly recommend that, in any 1H-MRS experiment, the MR acquisition parameters should be adjusted on a case dependent basis in order to obtain optimum spectral resolution and Cho SNR. 

##  Author's Contributions 

E. Kousi and I. Tsougos contributed equally to the paper.

## Figures and Tables

**Table 1:** Patient demographics, voxel size, 1H-MRS Choline presence, and histology results. Dash (—) indicates benign lesions.

Patient	Age	Voxel size before contrast administration (cm^3^)	Voxel size after contrast administration (cm^3^)	tCho presence before contrast administration	tCho presence after contrast administration	Histopathological findings
1	33	3.375	3.375	No	No	—
2	50	3.375	3.375	No	No	—
3	74	8.000	7.500	Yes	Yes	Lobular adenocarcinoma
4	54	5.800	5.800	No	No	Invasive ductal carcinoma
5	60	8.000	8.000	Yes	Yes	Malignant phyllodes yumour
6	67	8.000	8.000	No	Yes	Infiltrative lobular adenocarcinoma
7	58	3.375	3.375	No	No	Grade II adenocarcinoma
8	43	4.900	4.900	No	No	—
9	49	3.700	3.700	Yes	Yes	Grade III adenocarcinoma
10	49	3.700	3.700	No	Yes	Grade III adenocarcinoma
11	67	3.375	3.375	No	No	—
12	35	3.375	4.160	Yes	Yes	—
13	35	3.375	4.115	Yes	No	**—**
14	35	3.375	3.375	No	No	—
15	52	8.000	8.000	No	No	—
16	47	4.500	4.500	Yes	Yes	Grade III adenocarcinoma
17	47	3.375	3.375	Yes	Yes	Grade III adenocarcinoma
18	63	6.380	3.375	Yes	Yes	Grade III adenocarcinoma
19	45	8.000	8.000	No	No	—
20	71	3.375	4.300	No	Yes	Lobular adenocarcinoma
21	53	6.500	6.500	No	No	Invasive ductal carcinoma
22	53	5.380	5.380	No	No	—
23	50	6.000	3.670	No	No	—
24	64	8.000	8.000	No	No	—
25	50	5.800	5.800	No	No	—
26	37	3.375	6.500	No	Yes	Infiltrative adenocarcinoma
27	52	3.375	3.700	No	Yes	Grade III adenocarcinoma

**Table 2:** Measures of overall accuracy, sensitivity, and specificity using tCho presence in discrimination of benign from malignant breast lesions.

tCho presence	(%) Accuracy	(%) Sensitivity	(%) Specificity
Before contrast injection	62.9	42.8	84.6
After contrast injection	85.1	78.5	92
